# Dietary Aluminium Exposure and Human Health: Sources, Bioavailability, Toxicokinetics, and Health Risk Assessment

**DOI:** 10.3390/nu18162719

**Published:** 2026-08-20

**Authors:** Łukasz Kogut, Czesław Puchalski, Julia Jastrzębska, Grzegorz Zaguła

**Affiliations:** 1 Department of Bioenergetics, Food Analysis and Microbiology, Institute of Food Technology and Nutrition, Faculty of Technology and Life Science, University of Rzeszów, Aleja Rejtana 16C, 35-959 Rzeszów, Poland; lukasz.kogut2@wp.pl (Ł.K.); cpuchal@ur.edu.pl (C.P.); 2 Medical Centre in Łańcut LLC, Ignacego Paderewskiego 5, 37-100 Łańcut, Poland; j.jastrzebska2105@gmail.com

**Keywords:** aluminium, dietary exposure, bioavailability, food contamination, drinking water, toxicokinetics, gut microbiota, gut–brain axis, health risk assessment, food safety

## Abstract

**Background/Objectives**: Aluminium is a widespread environmental element and food contaminant to which the general population is continuously exposed, primarily through diet and drinking water. Although gastrointestinal absorption is generally low, bioavailability varies according to chemical form, food matrix, and interactions with dietary components. Prolonged exposure can nevertheless result in gradual tissue accumulation. This review summarises current evidence on dietary aluminium exposure, factors influencing its bioavailability, toxicokinetics, biological effects, gut microbiota interactions, and population-level health risk. **Methods**: A comprehensive narrative literature review was conducted using publications retrieved from PubMed/MEDLINE, Scopus, Web of Science, and Google Scholar. Original research articles, review papers, and reports issued by international organisations were critically evaluated with particular emphasis on dietary sources, drinking water, food additives, food contact materials, gastrointestinal absorption, toxicokinetics, biological mechanisms, gut microbiota, and health risk assessment. **Results**: Food represents the principal source of aluminium exposure in the general population, while drinking water usually contributes a smaller but continuous fraction of total oral intake. Dietary exposure varies substantially between populations and is influenced by food composition, processing practices, the use of aluminium-containing additives, and migration from food contact materials. Aluminium bioavailability is modified by chemical speciation and dietary constituents, including citrate, phosphates, silicates, phytates, polyphenols, and essential minerals. Despite limited absorption, prolonged exposure can lead to gradual aluminium accumulation, particularly in bone tissue and the central nervous system. Proposed biological mechanisms include oxidative stress, mitochondrial dysfunction, disruption of mineral homeostasis, and inflammatory signalling. Emerging evidence also indicates that aluminium may alter the gut microbiota, impair intestinal barrier integrity, and influence the gut–brain axis. Population exposure assessments show considerable regional variation, with some groups approaching or exceeding established tolerable weekly intake values. **Conclusions**: Dietary aluminium exposure represents a relevant issue in nutritional toxicology and food safety. Although current evidence does not establish that typical dietary exposure directly causes chronic disease, long-term exposure, differences in bioavailability, and the possibility of elevated intake in selected population groups justify continued monitoring and further prospective human studies. Future research should integrate dietary intake, aluminium speciation, nutritional status, biomarkers of internal exposure, and long-term health outcomes to improve risk assessment and support effective exposure-reduction strategies.

## 1. Introduction

Aluminium (Al) is the most abundant metal in the Earth’s crust and the third most abundant element after oxygen and silicon [1,2,3,4,5,6]. It occurs naturally predominantly in mineral compounds and, although widely distributed in the environment, has no known essential physiological function in humans [3,7,8,9,10,11]. The extensive industrial and technological use of aluminium has increased the diversity of potential human exposure sources, including applications in construction, transportation, food packaging, electronics, and medicine [1,2,3,4,5,6,8,12].

For the general population, diet represents the principal source of continuous aluminium exposure [3,9,13,14,15]. Aluminium may originate from its natural presence in foods, aluminium-containing food additives, drinking water, and migration from food contact materials [1,3,8,11,13,16,17]. Non-dietary sources, including pharmaceuticals, cosmetics, and selected medical products, may additionally contribute to total exposure, although food and drinking water remain particularly relevant to chronic oral exposure [1,2,11,13].

The potential biological significance of dietary aluminium cannot be assessed solely on the basis of total intake because gastrointestinal bioavailability depends on chemical speciation and interactions with dietary constituents [18,19,20]. Gastrointestinal absorption is generally very low, with estimates of approximately 0.1% for aluminium ingested with food and approximately 0.3% for aluminium present in drinking water [3]. Nevertheless, continuous exposure permits the systemic uptake of small amounts of aluminium, which may subsequently be distributed to and retained in several tissues [3,6,9,14,21,22,23]. Consequently, chronic exposure, even at comparatively low doses, has raised questions regarding the potential significance of gradual aluminium accumulation [18,21].

Despite decades of research, the health consequences of chronic low-dose aluminium exposure remain incompletely understood. Adverse effects associated with high exposure, including particular occupational and clinical scenarios, are better established than those resulting from typical long-term dietary exposure [5,19,24]. Interpretation is further complicated by differences in exposure sources, aluminium bioavailability, dietary composition, nutritional status, and other individual or environmental factors [9,13,25]. Moreover, much of the available evidence derives from experimental or cross-sectional studies, highlighting the need for stronger prospective human evidence [12,13,19].

Recent literature has extensively addressed individual aspects of aluminium exposure, including dietary sources, bioavailability, toxicokinetics, and neurotoxicity [16,19]. However, an important unresolved issue is how these different levels of evidence should be integrated when evaluating the potential health significance of chronic dietary exposure. Total aluminium intake alone provides an incomplete measure of biologically relevant exposure because gastrointestinal bioavailability depends on chemical speciation and interactions with dietary constituents, while subsequent systemic exposure is influenced by aluminium distribution and retention [18,19,20,26]. Moreover, the interpretation of potential health effects remains complicated by substantial differences in exposure conditions and by the predominance of experimental and cross-sectional evidence over prospective human studies [12,13,19]. Therefore, rather than treating dietary exposure, bioavailability, toxicokinetics, biological mechanisms, and health outcomes as separate issues, the present review integrates these elements within an exposure-to-effect framework linking external dietary exposure with gastrointestinal bioavailability, internal aluminium burden, mechanistic plausibility, and the strength of evidence for potential health effects. Gut microbiota and gut–brain interactions are incorporated into this framework as potential components of the biological response to aluminium exposure rather than being treated as an independent source of novelty. This integrative approach aims to clarify the current disconnect between estimates of external dietary aluminium exposure and their biological and toxicological relevance, while identifying the major uncertainties that limit interpretation of long-term dietary exposure and the identification of potentially susceptible population groups.

**Figure 1:**
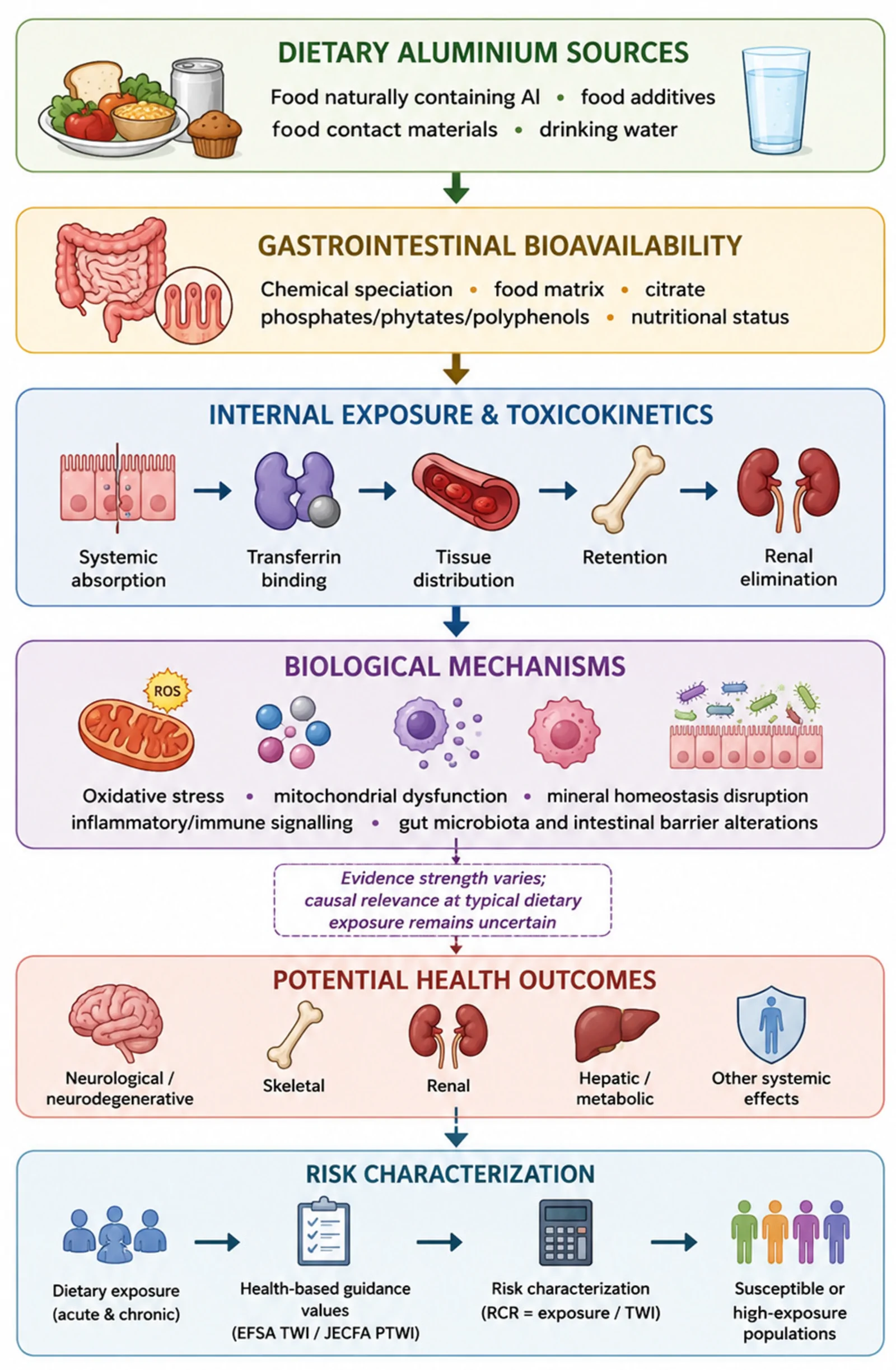
Integrative exposure-to-effect framework for dietary aluminium. Source: Authors’ own elaboration. The figure is original and does not contain third-party copyrighted material.

The integrative exposure-to-effect framework adopted in the present review is summarised in Figure 1, linking external dietary aluminium exposure with gastrointestinal bioavailability, internal aluminium burden, biological mechanisms, potential health outcomes, and population-level risk characterisation.

## 2. Materials and Methods

The literature search focused primarily on publications from 2000 to 2026, with particular emphasis on recent studies addressing dietary aluminium exposure, its major food and drinking-water sources, bioavailability, toxicokinetics, and potential health effects associated with chronic exposure. Selected earlier studies and authoritative reports were also included when they provided foundational toxicokinetic, mechanistic, or regulatory information relevant to the scope of the review. The literature search was conducted using Boolean operators (“AND”, “OR”) in combination with the following keywords: aluminum, aluminium, dietary exposure, dietary intake, food contamination, food additives, food contact materials, drinking water, bioavailability, gastrointestinal absorption, food matrix, dietary factors, toxicokinetics, bioaccumulation, oxidative stress, mitochondrial dysfunction, gut microbiota, gut–brain axis, mineral homeostasis, chronic exposure, health risk assessment, and human health. Reference lists of relevant publications were also consulted to identify additional literature pertinent to the scope of the review.

The literature considered in this narrative review comprised original research articles, review papers, and reports from international organisations and regulatory authorities, including EFSA and WHO/JECFA. Priority was given to full-text publications in English or Polish that provided information relevant to chronic aluminium exposure, particularly from dietary sources and drinking water, as well as to bioavailability, toxicokinetics, mechanisms of action, and potential health effects. Conference abstracts without full-text availability, editorials, conference proceedings, and publications not directly relevant to the scope of the review were generally not considered.

The identified literature was critically evaluated and synthesised according to the objectives of this narrative review. The analysis focused on dietary sources of aluminium, food additives and food contact materials, drinking-water exposure, factors affecting gastrointestinal absorption and bioavailability, systemic transport, bioaccumulation and elimination, biological mechanisms of toxicity, potential health effects, interactions with the gut microbiota, and regional patterns of dietary exposure and population-level health risk assessment. Particular attention was given to the influence of dietary factors and nutritional status on aluminium bioavailability, the relevance of experimental findings to typical human dietary exposure, and population groups potentially at increased risk of excessive exposure. Because the review integrates heterogeneous evidence from human observational studies, experimental animal models, in vitro studies, exposure assessments, toxicokinetic investigations, and regulatory reports, the evidence was interpreted according to study type, exposure conditions, biological relevance, and applicability to chronic dietary exposure in humans. Greater interpretative weight was assigned to human data and authoritative exposure or risk assessments when evaluating population-level health relevance, whereas experimental studies were primarily used to assess mechanistic plausibility. Particular attention was given to consistency between studies, differences in aluminium dose and route of exposure, correspondence between experimental and dietary-relevant exposure levels, and major methodological limitations affecting extrapolation to humans. Evidence derived predominantly from experimental models or from exposure conditions substantially exceeding typical dietary intake was therefore distinguished from evidence directly obtained in human populations.

No formal numerical quality score, risk-of-bias instrument, or evidence-grading system was applied, as these tools were not used prospectively for study selection and the heterogeneous evidence base did not permit application of a single appraisal framework across all included source types. A formal meta-analysis or pooled effect estimate was not performed because the included evidence was highly heterogeneous with respect to study design, study population, aluminium species, exposure route, dose, exposure duration, biological matrix, and reported outcome measures, precluding the derivation of a single statistically meaningful summary effect. Quantitative synthesis was therefore restricted to directly comparable exposure metrics and health-based guidance values, whereas heterogeneous mechanistic and health-outcome evidence was integrated using a structured critical appraisal framework considering evidence type, consistency, methodological limitations, and relevance to chronic dietary exposure. Given the narrative nature of the review, study selection and synthesis were not conducted according to a formal systematic-review protocol or PRISMA framework. As the study did not involve human participants or experimental procedures, ethics committee approval was not required.

## 3. Dietary Sources and Determinants of Aluminium Exposure

Human exposure to aluminium is widespread and originates from both its natural occurrence in the environment and its extensive use in industrial and technological processes [26,27,28,29,30,31]. As the third most abundant element in the Earth’s crust and the most abundant metal, aluminium is naturally present in soil, water, air, and living organisms [26,31]. For the general population, food and drinking water constitute the principal sources of continuous exposure, although pharmaceuticals, cosmetics, and occupational exposure may substantially increase aluminium intake in specific population groups [26]. The amount of aluminium entering the body, its bioavailability, and its potential biological significance vary considerably depending on the source and route of exposure [31].

### 3.1. Natural Occurrence of Aluminium in Foods

Food is considered the principal source of aluminium exposure for the general population, whereas drinking water generally contributes a substantially smaller proportion of total oral intake [26]. Aluminium present in food may be of natural, environmental, or technological origin, and its concentration depends on the type of food, cultivation conditions, degree of processing, and methods of storage and preparation [26,30,32].

The agricultural environment plays an important role in determining the aluminium content of plant-derived foods because plants may take up aluminium ions from soil and water [26]. Aluminium bioavailability in soil increases markedly under acidic conditions, which may result from industrial activity, atmospheric pollution, and acid precipitation [20,26]. A decrease in soil pH promotes the transformation of aluminium from poorly soluble mineral forms into more soluble ionic species that may become more available for plant uptake [33]. Certain plant-derived foods may contain substantially higher aluminium concentrations than many commonly consumed unprocessed foods [16,26]. According to the comprehensive compilation by Yokel, tea leaves contain particularly high aluminium concentrations, with a median of approximately 805 mg/kg, compared with approximately 225 mg/kg in herbal teas [16]. Cocoa beans also contain elevated amounts, with a reported median concentration of approximately 55.6 mg/kg [16]. Aluminium concentrations in spices and aromatic herbs are highly variable, with values reported in the literature ranging from several mg/kg to several tens of mg/kg, and some products showing substantially higher concentrations [16]. In comparison, many ordinary unprocessed foods contain aluminium in the low mg/kg range; for example, the median concentration reported for fruits is approximately 1.1 mg/kg [16]. Thus, on a mass basis, tea leaves and certain herbs, spices, and cocoa products may contain aluminium concentrations one to several orders of magnitude higher than many commonly consumed unprocessed foods. However, their contribution to total dietary exposure also depends strongly on the amount consumed and, in the case of tea and herbal products, on the fraction of aluminium transferred into the prepared infusion [16,26].

### 3.2. Aluminium-Containing Food Additives

Food additives and technological agents containing aluminium may contribute substantially to total dietary exposure [26,34]. Aluminium compounds have been used as leavening agents, anti-caking agents, stabilisers, firming agents, and colourants [34]. These include aluminium sulphates (E520–E523), sodium aluminium phosphate, acidic (E541), sodium aluminium silicate (E554), and potassium aluminium silicate (E555). Other aluminium-containing silicates, including calcium aluminium silicate (E556), bentonite (E558), and aluminium silicate (kaolin; E559), were previously authorised for food use in the European Union but their authorised food uses were withdrawn following regulatory restrictions introduced to reduce dietary aluminium exposure. Aluminium metal (E173) is also authorised as a food colour, although its use is restricted to specific decorative applications. The principal aluminium-containing food additives, their technological roles, and current regulatory status are summarised in Table 1.

Elevated aluminium concentrations have been reported in bakery products, confectionery, biscuits, baking mixes, and certain types of pasta and cereal-based products [33,34]. In some cases, technological processing rather than the natural aluminium content of the raw materials may therefore be responsible for elevated concentrations in the final food product [26,35,36,37]. From a global perspective, increasing industrialisation of agriculture and food production has contributed to the growing importance of anthropogenic sources of aluminium in the human diet [26,37]. Contemporary dietary exposure therefore reflects not only the natural occurrence of aluminium in the environment but also its introduction throughout different stages of the food production chain [33,37].

### 3.3. Food Processing and Food Contact Materials

Migration from food contact materials represents an additional source of dietary aluminium exposure [26,28,30]. Aluminium is widely used in the manufacture of cookware, frying pans, trays, foil, and beverage and food containers [28,30]. The migration of aluminium ions into food depends on several factors, including temperature, duration of contact, salt content, and food pH [26,30].

Migration may be particularly pronounced during the preparation or storage of acidic and saline foods, including tomato-based products, fruits, marinades, and fermented foods [28]. Although modern metal packaging is generally protected by internal coatings that limit direct contact between food and aluminium, damage to these protective layers or inappropriate storage conditions may increase aluminium release into food products. Therefore, food preparation, processing, packaging, and storage conditions may influence dietary aluminium exposure independently of the natural aluminium content of the consumed food [26,36].

### 3.4. Aluminium in Drinking Water and Beverages

Drinking water represents an additional continuous source of oral aluminium exposure [26,38]. Although its contribution to total daily intake is generally substantially lower than that of food, aluminium dissolved in water may exhibit relatively high bioavailability, potentially increasing its biological significance [20,26].

Aluminium occurs in both surface water and groundwater as a result of natural geochemical processes and anthropogenic activities [33,37]. Water treatment represents an additional source because aluminium salts, including aluminium sulphate and polyaluminium chloride, are widely used as coagulants and flocculants to remove suspended particles, organic matter, and microorganisms [26,38]. Small amounts of residual aluminium may, however, remain in drinking water following treatment [19,38].

Environmental conditions also influence aluminium concentrations in water. The acidification of soils and aquatic ecosystems increases the solubility of aluminium compounds and may consequently increase concentrations of dissolved aluminium in surface waters [38,39]. This phenomenon is particularly relevant in regions affected by intensive industrial emissions and acid precipitation [26,38,39].

In the European Union, aluminium is subject to a parametric value of 0.2 mg/L in drinking water. This value should be distinguished from the WHO approach, as WHO does not establish a formal health-based guideline value for aluminium in drinking water; instead, its recommendations focus on practicable operational control of residual aluminium concentrations during water treatment [38].

The potential significance of drinking water as a source of aluminium exposure also reflects differences in bioavailability. Aluminium dissolved in water may be more readily absorbed than aluminium present in many food matrices [26,40]. Gastrointestinal absorption has been estimated at approximately 0.1% for aluminium ingested with food and approximately 0.3% for aluminium present in drinking water [26]. Consequently, continuous consumption of drinking water containing even relatively low aluminium concentrations can add to long-term systemic exposure [26,38,41].

### 3.5. Non-Dietary Sources in the Context of Total Aluminium Exposure

Although dietary intake and drinking water constitute the principal sources of continuous aluminium exposure in the general population, non-dietary and medical sources may become dominant in specific circumstances. This distinction is important when assessing total body burden because exposure levels and routes may differ substantially from those associated with conventional dietary intake [26].

Pharmaceutical products containing aluminium, particularly antacid preparations, may provide substantially higher aluminium doses than those typically obtained from food [26,31]. A single dose may contain tens to hundreds of milligrams of aluminium, and chronic use may therefore markedly increase total exposure. Individuals with impaired renal function are particularly vulnerable because their capacity for aluminium elimination is reduced [26,42,43,44,45,46]. Aluminium contamination of parenteral nutrition solutions also represents an important clinical concern because contamination may originate from both manufacturing processes and packaging materials [42,45]. This issue is particularly relevant for preterm infants, neonates, and patients requiring long-term parenteral nutrition [42,44].

Additional sources of aluminium exposure include selected medicinal products, vaccine adjuvants, dental and implant materials, cosmetics, and personal care products [31,47,48,49]. Antiperspirants containing aluminium salts, particularly aluminium chlorohydrate, represent a source of dermal aluminium exposure, although absorption through intact skin appears to be very limited and may increase when the skin barrier is disrupted [40,49,50]. The potential relationship between long-term exposure to aluminium-containing antiperspirants and breast cancer has also been investigated; however, the available evidence remains inconclusive and does not establish a causal association [29,41].

Occupational exposure may additionally result in substantially higher aluminium doses than those encountered by the general population, particularly among workers employed in mining, smelting, metallurgy, welding, and related industries. In these settings, inhalation of aluminium-containing dust and aerosols represents the predominant exposure route [31]. Long-term occupational exposure is therefore recognised as an important risk factor for excessive aluminium accumulation. Nevertheless, these sources represent distinct exposure scenarios and should be differentiated from the chronic, low-dose oral exposure associated with food and drinking water, which remains the principal focus of the present review.

For quantitative context, EFSA estimated mean dietary aluminium exposure in European adults at approximately 1.6–13 mg/day, corresponding to about 0.2–1.5 mg/kg body weight/week [26]. Occupational exposure represents a fundamentally different exposure scenario because aluminium is predominantly inhaled as dust or aerosols rather than ingested [31]. Workers employed in mining, smelting, metallurgy, welding, and related industries may therefore experience substantially higher aluminium exposure than the general population [31]. However, occupational exposure cannot be directly expressed as an equivalent dietary intake in mg/day because the routes of exposure and associated absorption differ substantially [26,31]. Dietary intake and occupational exposure should therefore be considered distinct but potentially complementary contributors to total aluminium body burden [26,31].

## 4. Dietary Bioavailability and Factors Modifying Aluminium Absorption

For the general population, oral exposure through food and drinking water represents the principal route of aluminium exposure [26]. Aluminium enters the body primarily through food and drinking water, although certain aluminium-containing pharmaceutical products may result in substantially higher exposure in specific scenarios [26,31]. Despite continuous oral exposure, aluminium is characterised by relatively low gastrointestinal bioavailability. Nevertheless, repeated intake over prolonged periods may result in the entry of small amounts of aluminium into the systemic circulation and contribute to a gradual increase in the total body burden [51,52,53,54,55].

### 4.1. Gastrointestinal Absorption and Chemical Speciation

Aluminium absorption occurs primarily in the duodenum and proximal small intestine, although the efficiency of this process is generally low [53,56,57]. Gastrointestinal absorption of orally ingested aluminium is generally very low; EFSA estimated absorption at approximately 0.1% from food and approximately 0.3% from drinking water [26]. Despite this low absorption rate, chronic dietary exposure results in the continuous transfer of small amounts of aluminium into the circulation [53].

The bioavailability of aluminium is strongly influenced by its chemical form and solubility within the gastrointestinal tract [26,52]. Aluminium speciation varies according to pH and the presence of ligands capable of forming either soluble or poorly soluble complexes [56]. Under acidic conditions, aluminium is more likely to remain in soluble forms, which may increase its potential for gastrointestinal absorption [11,26]. In contrast, under neutral conditions aluminium readily forms poorly soluble hydroxides that are not efficiently absorbed and are subsequently eliminated with the faeces [53,57].

These physicochemical characteristics indicate that total aluminium intake alone does not fully determine systemic exposure. Instead, the fraction that becomes biologically available depends substantially on the chemical species present within the gastrointestinal environment and on interactions with components of the consumed food matrix [38,53,58].

### 4.2. Food Matrix and Dietary Factors Affecting Bioavailability

A number of dietary constituents may substantially modify aluminium solubility and gastrointestinal absorption [51,52]. Complexing agents that form soluble aluminium compounds can enhance its bioavailability. Citric acid and citrate salts are among the most important examples because they increase aluminium solubility in the gastrointestinal tract and may result in several-fold increases in absorption [53,59]. Similar effects have been reported for lactates and maltol [52].

Conversely, dietary compounds capable of forming poorly soluble aluminium complexes may reduce gastrointestinal absorption [52,53]. Phosphates, silicates, and phytates, which are naturally present in numerous foods, particularly plant-derived products, may limit aluminium bioavailability by decreasing its solubility in the intestinal environment [52]. Reduced absorption has also been observed in the presence of certain dietary polyphenols [51,52,59].

The composition of the food matrix may therefore substantially influence the amount of ingested aluminium that reaches the systemic circulation. This is particularly relevant when comparing aluminium exposure from different dietary sources, as identical total concentrations may not necessarily result in equivalent absorbed doses. The biological significance of dietary aluminium exposure should therefore be interpreted in the context of both total intake and the physicochemical characteristics of the ingested food [51,52].

Human volunteer data further demonstrate that aluminium bioavailability may differ according to its chemical form and source. Following ingestion of sodium aluminium phosphate (SALP), a food additive, increased urinary aluminium excretion was observed in healthy volunteers, confirming systemic absorption of additive-derived aluminium [60]. These findings indicate that aluminium from food additives may contribute to internal exposure and should not necessarily be considered toxicokinetically equivalent to aluminium naturally present in complex food matrices.

### 4.3. Interactions with Essential Minerals and Nutritional Status

Nutritional status may represent an additional determinant of aluminium absorption and retention. Deficiencies of essential minerals, particularly iron, calcium, and magnesium, may increase aluminium uptake through enhanced utilisation of shared or overlapping transport pathways [52,53].

The interaction between aluminium and iron is of particular importance because both elements may utilise common transport proteins and metabolic pathways. Aluminium can interfere with physiological iron handling, while low iron status may increase the biological availability of aluminium by enhancing the activity of mechanisms involved in metal uptake. Similar interactions have been proposed for calcium and magnesium, suggesting that inadequate nutritional status may increase susceptibility to aluminium absorption and subsequent accumulation [52,53].

These findings indicate that individual susceptibility to dietary aluminium exposure may depend not only on the amount consumed but also on the nutritional status of the exposed individual. Consequently, the assessment of aluminium-related health risks should take into account both dietary exposure and potential interactions with essential nutrients.

## 5. Toxicokinetics of Dietary Aluminium

Aluminium toxicokinetics encompasses the processes of systemic transport, tissue distribution, bioaccumulation, and elimination following absorption from the gastrointestinal tract [51,52]. Unlike essential trace elements, aluminium has no known physiological function, and its presence in human tissues results from environmental, dietary, occupational, or medical exposure [53,61]. A characteristic feature of aluminium is its low bioavailability combined with its capacity for prolonged retention in selected tissues [62,63,64,65,66,67,68,69,70]. Consequently, even relatively low absorbed doses may contribute to gradual accumulation when exposure is sustained over long periods or when elimination is impaired [71].

**Figure 2:**
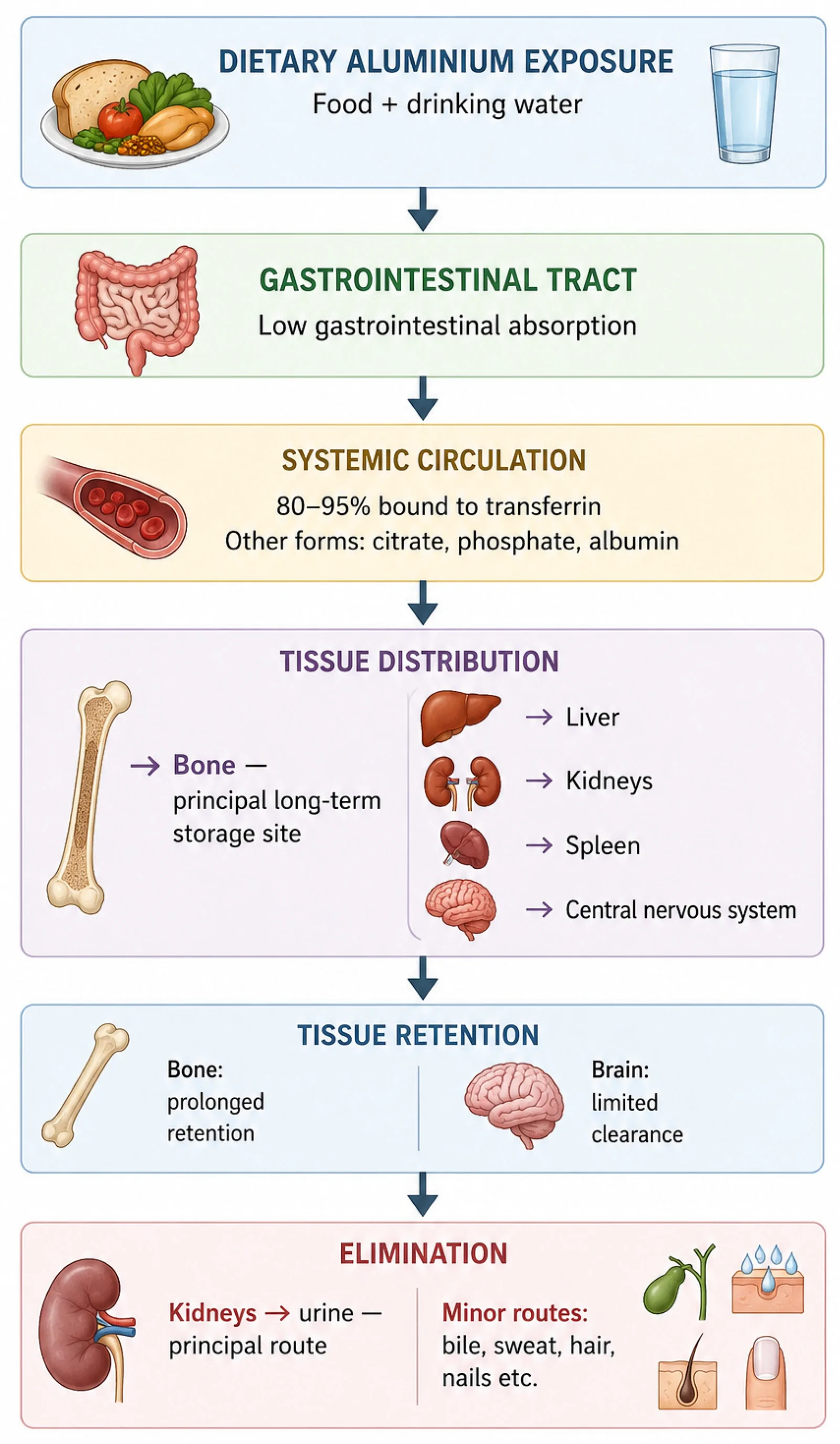
Schematic overview of the toxicokinetics of dietary aluminium, including gastrointestinal absorption, systemic transport, tissue distribution, long-term retention, and elimination. Source: Authors’ own elaboration. The figure is original and does not contain third-party copyrighted material.

The principal toxicokinetic processes governing dietary aluminium absorption, systemic transport, tissue distribution, accumulation, and elimination are summarised in Figure 2.

### 5.1. Systemic Transport and Distribution

Following intestinal absorption, aluminium enters the bloodstream and rapidly binds to plasma proteins [59,61]. The principal carrier is transferrin, the protein responsible for physiological iron transport [51,61,62,63]. Approximately 80–95% of circulating serum aluminium is estimated to be present in transferrin-bound complexes [53,62,63]. In healthy adult populations, mean circulating aluminium concentrations are generally in the low µg/L range. Mean plasma aluminium concentrations of 6.9 µg/L have been reported in healthy Australian adults, whereas a large French population study reported a mean concentration of 4.32 µg/L [63]. Reference values of <10 µg/L for serum aluminium have been proposed, although values may vary according to geographical region, dietary habits, occupational environment, lifestyle, and analytical methodology [63]. Approximately 80–95% of circulating aluminium is associated with transferrin [53,62,63]. The remaining fraction should not be interpreted as entirely free ionic Al^3+^, because circulating aluminium may also occur in complexes with citrate, phosphate, or albumin [26,53]. Therefore, the truly free ionic fraction represents only a minor component of circulating aluminium, while the non-transferrin-bound fraction comprises other protein-associated and low-molecular-weight aluminium species [26,53]. This interaction reflects the chemical similarities between aluminium and iron ions, allowing aluminium to utilise pathways normally involved in essential metal transport [61,63].

Low-molecular-weight aluminium complexes, particularly aluminium–citrate complexes, may facilitate systemic distribution of aluminium [57,58]. Through transferrin receptor-mediated mechanisms and other transport pathways, aluminium reaches numerous organs, where it may subsequently accumulate [52,58].

The largest proportion of aluminium is deposited in the skeletal system, which serves as the principal storage site for this metal [26,52]. Aluminium has also been detected in soft tissues, including the lungs, liver, kidneys, and spleen [52,53]. Its presence in the central nervous system is of particular toxicological interest [55]. Aluminium can cross the blood–brain barrier through transport mechanisms involving transferrin-bound and low-molecular-weight aluminium species [52,55]. Studies have identified aluminium accumulation particularly in the hippocampus and cerebral cortex, regions involved in memory, learning, and cognitive function [55,65].

### 5.2. Tissue Accumulation

One of the most distinctive features of aluminium toxicokinetics is its capacity for prolonged retention and tissue-specific accumulation [52,53,68]. The total body burden of aluminium in healthy individuals is low but may increase substantially during chronic exposure or when renal elimination is impaired [52,53]. This is particularly relevant in chronic kidney disease, where reduced renal clearance can lead to a several-fold increase in circulating aluminium compared with healthy individuals. Mean plasma concentrations of approximately 4.32–6.9 µg/L have been reported in healthy adults, whereas clinical guidance for haemodialysis patients uses substantially higher concentration thresholds, including <20 µg/L and, in some laboratory reference frameworks, <60 µg/L [63]. These values should not be interpreted as fixed mean concentrations in all patients with chronic kidney disease, but they illustrate the markedly greater systemic aluminium burden that may occur when renal elimination is impaired. Aluminium is not distributed uniformly throughout the body but shows a marked affinity for selected tissues [52,68].

In this context, tissue accumulation should not be interpreted simply as aluminium concentrations in individual organs being consistently higher than those measured in blood. Rather, accumulation reflects preferential tissue deposition combined with tissue-specific retention and slower clearance following repeated exposure [52,53,68,69]. Blood aluminium represents the circulating and comparatively dynamic compartment, whereas aluminium deposited in tissues may exhibit substantially longer residence times. This distinction is particularly evident from the marked differences in biological half-life between tissues: aluminium retention in the liver is generally less than two days, whereas retention in the kidneys and spleen may extend from several days to approximately two weeks [69,70]. In contrast, skeletal aluminium may have a biological half-life of approximately 7–20 years [67], while aluminium deposited in the central nervous system is characterised by limited clearance and may persist for prolonged periods [69,71]. Thus, the term “accumulation” primarily refers to progressive tissue retention when repeated uptake exceeds tissue-specific elimination rather than to a universally higher aluminium concentration in tissues than in blood.

As noted above, skeletal deposition is a major component of aluminium distribution. Under conditions of elevated exposure or impaired aluminium elimination, prolonged retention at bone mineralisation surfaces interferes with normal bone mineralisation and can contribute to osteomalacia [26,52,61]. Aluminium deposition in nervous tissue is also considered toxicologically relevant [53,55]. Studies have detected aluminium in neuronal and glial compartments as well as in cerebral blood vessels [55,65].

### 5.3. Elimination and Factors Affecting Retention

Renal excretion represents the principal route of aluminium elimination from the human body [52,53]. In individuals with normal renal function, absorbed aluminium is eliminated predominantly through urinary excretion [26,52]. Renal elimination primarily involves the unbound fraction and low-molecular-weight aluminium complexes, including aluminium–citrate complexes [52,57]. Renal function therefore plays a critical role in maintaining the balance between aluminium uptake, retention, and elimination [26,52].

Urinary aluminium may be used as a biomarker of internal aluminium exposure because renal excretion represents the principal elimination route for absorbed aluminium [26,52,53]. However, urinary aluminium concentration should not be interpreted as a direct quantitative measure of total aluminium exposure or body burden. Urinary excretion reflects the fraction of aluminium that has entered the systemic circulation and is available for renal elimination, and is therefore influenced by gastrointestinal absorption, aluminium speciation, systemic distribution, tissue retention, and renal function [26,52,53,57]. Consequently, urinary aluminium measurements may be useful for identifying increased recent or ongoing systemic exposure, particularly when exposure is relatively high, but their ability to reconstruct chronic dietary intake is limited. This limitation is especially important in individuals with impaired renal function, in whom reduced aluminium clearance may increase systemic and tissue aluminium burden without a proportional increase in urinary aluminium excretion [52,53,63]. Thus, urinary aluminium should be interpreted together with exposure history, renal function, and, where appropriate, circulating aluminium concentrations rather than as a stand-alone quantitative marker of chronic dietary exposure.

Smaller amounts of aluminium may be eliminated through bile, sweat, hair, nails, breast milk, and semen [52,68]. However, most aluminium present within the gastrointestinal tract is never absorbed and is eliminated directly in the faeces [26,52]. From a biological perspective, this means that only a small proportion of ingested aluminium reaches the systemic circulation [62].

The efficiency of aluminium elimination may be reduced by several factors [52,53]. Strong binding to transferrin may limit glomerular filtration and prolong the residence time of aluminium in the circulation [52,53]. The greatest risk of excessive aluminium accumulation occurs in individuals with chronic kidney disease, in whom renal excretory capacity is substantially impaired [52,53]. Newborns, particularly preterm infants with immature renal function, also represent a vulnerable population [62].

Patients undergoing haemodialysis represent a distinct high-risk population because reduced renal clearance may be combined with additional aluminium exposure from dialysis-related sources. In healthy adults, mean plasma aluminium concentrations of approximately 4.32–6.9 µg/L have been reported, with serum reference values generally below 7–10 µg/L [63]. In contrast, reference values used for dialysis patients are substantially higher; Mayo Clinic guidance cited by Pérez San Martín et al. indicates a serum aluminium reference value below 60 µg/L in dialysis patients, whereas the Spanish Society of Nephrology recommends maintaining blood aluminium concentrations below 20 µg/L in patients receiving haemodialysis [63]. Thus, circulating aluminium concentrations in dialysis-related clinical settings may be several-fold higher than those typically observed in healthy populations. However, these values should not be interpreted as a fixed fold-increase in exposure, because aluminium burden varies according to residual renal function, dialysis-water contamination, use of aluminium-containing medications, and historical versus contemporary dialysis practices [63].

Parenteral nutrition constitutes a distinct clinical exposure scenario because aluminium administered with parenteral nutrition bypasses the gastrointestinal barrier and enters directly into the systemic circulation [59,67,72]. Under these conditions, even relatively small amounts of aluminium may contribute to rapid systemic accumulation and increase the risk of toxic effects [59,67,72]. This route should therefore be distinguished from conventional dietary exposure, where gastrointestinal absorption strongly limits the fraction of aluminium reaching the systemic circulation [1,59,67,72].

## 6. Biological Mechanisms Linking Chronic Aluminium Exposure to Health Effects

The mechanistic evidence discussed in this section should be interpreted in the context of the experimental conditions under which it was obtained. Much of the current evidence for aluminium-induced oxidative stress, mitochondrial dysfunction, disruption of mineral homeostasis, inflammatory signalling, and cellular damage derives from in vitro studies and animal models using exposure levels that may substantially exceed those typically encountered through food and drinking water [6,73,74,75]. Consequently, these findings demonstrate biologically plausible mechanisms of aluminium toxicity but should not be interpreted as evidence that the same effects necessarily occur in humans at typical dietary exposure levels. Evidence directly demonstrating these mechanisms under chronic, dietary-relevant exposure conditions remains comparatively limited [6,26].

Aluminium toxicity is multifactorial and involves disturbances at the molecular, cellular, and organ levels [6]. As aluminium has no known essential physiological function, its accumulation in the human body may interfere with fundamental metabolic processes [73,74]. Although particular attention has been directed toward its neurotoxic potential, the biological effects of aluminium involve several interconnected mechanisms, including oxidative stress, mitochondrial dysfunction, disturbances of mineral homeostasis, activation of inflammatory pathways, and adverse interactions with proteins, nucleic acids, and cellular structures [75,76].

### 6.1. Oxidative Stress and Mitochondrial Dysfunction

Oxidative stress is considered one of the principal mechanisms underlying aluminium toxicity [23,74]. Although Al^3+^ is not a classical redox-active metal, it may indirectly promote reactive oxygen species (ROS) formation by disturbing iron-dependent processes and increasing the availability of redox-active iron, thereby facilitating Fenton-type reactions [73,75,77,78,79,80]. Aluminium may also interact with superoxide species, promoting the formation of hydrogen peroxide and hydroxyl radicals. Experimental studies consequently report lipid peroxidation, protein carbonylation, and oxidative DNA damage, including increased 8-hydroxy-2′-deoxyguanosine concentrations [6,74]. However, the magnitude of aluminium-induced disruption of iron homeostasis depends strongly on dose and biological context, and its occurrence at typical dietary exposure levels in humans has not been established [6,63,74,75].

Aluminium exposure may additionally impair endogenous antioxidant defence. Experimental studies have reported reductions in glutathione levels and in the activities of superoxide dismutase, catalase, glutathione peroxidase, and glutathione reductase [75,79]. Alterations in Nrf2/Keap1-dependent antioxidant signalling have also been described and may contribute to reduced cellular antioxidant capacity and increased susceptibility to ROS-mediated damage [75,81]. Nevertheless, the precise interaction between aluminium and this signalling pathway remains incompletely characterised and is supported predominantly by experimental evidence.

Mitochondria represent another important target of aluminium toxicity [74,82,83]. Aluminium may impair enzymes of the tricarboxylic acid cycle and mitochondrial electron transport, leading to reduced membrane potential, decreased ATP production, and increased electron leakage and ROS generation [6,74,75,82,83,84,85]. This creates a self-reinforcing relationship between oxidative stress and mitochondrial dysfunction. Experimental studies have also reported compensatory increases in glycolytic metabolism, alterations in HIF-1α signalling, and impaired mitochondrial biogenesis involving the PGC-1α/NRF-1/Tfam pathway [82,83,84]. Morphological abnormalities, including mitochondrial swelling and disruption of cristae, have also been observed [6,74].

Overall, oxidative stress and mitochondrial dysfunction constitute well-supported mechanisms of aluminium toxicity under experimental conditions. However, much of this evidence derives from animal and in vitro studies using exposure levels higher than those typically encountered through the human diet. Direct evidence confirming these mechanisms during chronic dietary-relevant aluminium exposure in humans therefore remains limited [6,74,75].

### 6.2. Disruption of Mineral Homeostasis

Another important mechanism of aluminium toxicity is its ability to interfere with the homeostasis of essential mineral elements [23,83,85,86,87]. Owing to its high charge-to-radius ratio, Al^3+^ can compete with other biologically important cations for binding sites in proteins, enzymes, and cellular structures [23,86]. Interactions with iron, calcium, and magnesium are considered particularly relevant [23,82,87].

In the case of iron, aluminium may exploit its chemical similarity to Fe^3+^ and bind to transferrin and ferritin, thereby interfering with normal iron transport and storage [6,81]. Experimental evidence has also suggested that aluminium may affect iron-regulatory proteins, including stabilization of IRP2, potentially increasing transferrin receptor expression and reducing ferritin synthesis [23,73]. Such alterations may increase the intracellular pool of labile iron and promote redox reactions and ROS formation [74,82]. Aluminium-associated disturbances in iron metabolism have consequently been proposed to increase susceptibility to ferroptosis, an iron-dependent form of regulated cell death associated with lipid peroxidation [75,79]. However, these specific molecular events have been characterised predominantly in experimental models, and their reproducibility and relevance to chronic dietary aluminium exposure in humans remain insufficiently established.

Aluminium may also disturb calcium homeostasis [6,83]. It can interfere with calcium-binding sites, voltage-dependent calcium channels, and calmodulin-dependent Ca^2+^/Mg^2+^-ATPase activity [23,73]. The resulting increase in intracellular calcium may promote excessive activation of calcium-dependent enzymes, impair neuronal signalling, contribute to excitotoxicity, and activate apoptotic pathways [73,75].

Interactions with magnesium are also biologically relevant. Al^3+^ has a strong affinity for phosphate groups and may compete with Mg^2+^ in ATP-dependent reactions [6,86]. This may interfere with the activity of magnesium-dependent enzymes, including kinases, ATPases, and glycolytic enzymes. Collectively, these interactions indicate that aluminium toxicity is closely linked to disturbances in mineral metabolism, which may be particularly relevant under conditions of chronic exposure and inadequate nutritional status [73,86,88].

Evidence for aluminium-induced disruption of mineral homeostasis is derived predominantly from experimental and mechanistic studies. Although interactions of aluminium with iron-, calcium-, and magnesium-dependent processes provide biologically plausible pathways of toxicity, their magnitude and clinical relevance under typical chronic dietary exposure conditions in humans remain insufficiently characterised [6,23,83,86,87].

### 6.3. Inflammation and Cellular Dysfunction

Experimental evidence indicates that aluminium-induced neuroinflammation involves activation of glial cells, particularly microglia and astrocytes [78,85]. Aluminium exposure has been associated with P2X7 receptor-dependent activation of the NLRP3 inflammasome and caspase-1-mediated maturation of IL-1β, as well as activation of NF-κB and increased production of pro-inflammatory mediators, including TNF-α, IL-1β, and IL-6 [6,23,76,85]. Activated microglia and astrocytes may further amplify inflammatory responses through the release of cytokines and reactive oxygen species [23,75,78]. Increased cyclooxygenase activity and systemic inflammatory markers, including C-reactive protein, have also been reported experimentally [23,75,81,88]. However, these inflammatory mechanisms have been demonstrated predominantly in experimental models, and their relevance to chronic dietary aluminium exposure in humans remains insufficiently established [23,85].

Additional molecular alterations have been proposed, including changes in inflammation-related microRNAs such as miR-9, miR-125b, and miR-146a [23,75,77]. Aluminium may also interact with proteins, nucleic acids, and cytoskeletal structures [23]. Experimental studies have reported abnormal protein aggregation, including β-amyloid oligomerization and tau hyperphosphorylation, as well as disturbances in microtubule and neurofilament organisation and impaired axonal transport [6,23,73,75,77,81,85]. However, these observations should not be interpreted as evidence that dietary aluminium causes Alzheimer’s disease or other neurodegenerative disorders, as their reproducibility and relevance to typical human dietary exposure remain uncertain.

Potential genomic and epigenetic effects have also been described, including interactions with DNA and chromatin, altered transcriptional accessibility, DNA methylation, and histone modifications [73,81,88,89]. These findings remain predominantly experimental and should therefore be regarded as emerging rather than firmly established mechanisms of aluminium toxicity.

Taken together, oxidative stress, mitochondrial dysfunction, altered mineral homeostasis, inflammatory signalling, and direct cellular interactions may converge on pathways leading to impaired cellular homeostasis and regulated cell death [74,90]. However, detailed molecular mechanisms such as inflammasome activation, microRNA alterations, abnormal protein aggregation, cytoskeletal disruption, and epigenetic changes remain supported primarily by experimental evidence. Their occurrence and clinical relevance under typical chronic dietary aluminium exposure in humans have not been established [6,23,74,75,77,81,88].

## 7. Potential Health Consequences of Chronic Dietary Aluminium Exposure

Chronic aluminium exposure has been associated with adverse effects across multiple organ systems, although the strength of evidence varies considerably depending on the exposure level, route, study design, and investigated health outcome. Much of the current mechanistic evidence derives from experimental models or populations exposed to substantially higher aluminium levels than those typically encountered through the diet. Consequently, the potential health effects of chronic dietary exposure should be interpreted cautiously, particularly when extrapolating experimental findings to the general population [8,91,92].

### 7.1. Neurological and Cognitive Effects

The nervous system is considered one of the principal targets of aluminium toxicity [8,91]. As described in Section 5.2, aluminium can reach and persist within the central nervous system. From a toxicological perspective, prolonged aluminium accumulation in nervous tissue may disturb cellular homeostasis and promote oxidative stress, inflammatory responses, and mitochondrial dysfunction, ultimately impairing neuronal function [91,92].

In contrast to the uncertainties surrounding typical dietary exposure, severe neurotoxicity associated with high systemic aluminium exposure is well established. Historically, aluminium accumulation in patients with impaired renal elimination, particularly those undergoing long-term dialysis, has been linked to dialysis encephalopathy, a clinically recognised neurological syndrome associated with excessive aluminium burden [6].

Particular attention has been directed toward the potential association between aluminium exposure and neurodegenerative diseases, especially Alzheimer’s disease [93]. Experimental evidence indicates that aluminium may promote β-amyloid aggregation and tau hyperphosphorylation, processes associated with the formation of amyloid plaques and neurofibrillary tangles. As discussed in the Introduction, the causal significance of these observations for Alzheimer’s disease remains unresolved; therefore, these findings should be interpreted as evidence of mechanistic plausibility rather than proof of a causal relationship [6,93].

Chronic aluminium exposure in occupationally and environmentally exposed populations has also been associated with impaired cognitive and neurological function [6]. Studies involving occupationally and environmentally exposed populations have reported alterations in memory, attention, information-processing speed, and motor coordination. These disturbances may be accompanied by chronic fatigue, irritability, learning difficulties, and reduced quality of life [6,92]. Experimental and observational evidence has further suggested that long-term aluminium accumulation may contribute to accelerated brain ageing and increased susceptibility to neurodegenerative processes, although the clinical significance of typical dietary exposure levels remains insufficiently established [6,91,92].

Taken together, the evidence for aluminium neurotoxicity is considerably stronger under conditions of high systemic exposure than for chronic dietary exposure in the general population. Experimental studies provide mechanistic plausibility through oxidative stress, mitochondrial dysfunction, neuroinflammation, and effects on amyloid- and tau-related pathways; however, their direct relevance to habitual dietary exposure is limited by differences in dose, aluminium species, route, and duration of exposure [6,23,75,81,85,91,92,93]. Epidemiological evidence concerning Alzheimer’s disease remains less conclusive, particularly because observational associations cannot by themselves establish causality and because exposure assessment and potential confounding complicate interpretation [8,91,93]. Accordingly, the available evidence supports the neurotoxic potential of aluminium, particularly under elevated-exposure conditions, but does not establish a causal relationship between typical dietary aluminium exposure and Alzheimer’s disease [6,8,91,93].

### 7.2. Bone and Mineral Metabolism

As described in Section 5.2, prolonged skeletal retention of aluminium has well-established toxicological consequences under conditions of high exposure or impaired renal elimination. Aluminium accumulation at sites of active mineralization interferes with calcium and phosphate metabolism, impairs osteoblast function, and suppresses new bone formation [6,8]. Clinically significant aluminium accumulation can cause impaired bone mineralization and osteomalacia, particularly in patients with renal failure or historically high aluminium exposure [6,94,95]. Reduced mineralization decreases bone strength and increases susceptibility to skeletal deformities and fractures [94,95]. The strongest clinical evidence derives from patients undergoing long-term dialysis, in whom aluminium overload has been associated with adynamic bone disease and markedly reduced bone turnover [6,94,95].

Aluminium may additionally interfere with the hormonal regulation of calcium and phosphate homeostasis. Accumulation within the parathyroid glands has been associated with impaired parathyroid hormone secretion, potentially contributing to further disturbances in bone mineralization and skeletal metabolism. However, evidence concerning the extent to which chronic exposure at typical dietary levels affects bone health in individuals with normal renal function remains limited [6].

### 7.3. Haematological Effects

Chronic aluminium exposure, particularly under conditions of elevated systemic aluminium burden, is associated with haematological abnormalities [6,92]. One of the most consistently described effects is microcytic anaemia, which can respond poorly to conventional iron supplementation [6,94]. The underlying mechanisms are primarily associated with disturbances in iron metabolism and impaired haemoglobin synthesis [6,21].

As described in Section 6.2, aluminium-induced disruption of iron homeostasis may reduce iron availability for erythropoiesis and thereby contribute to haematological abnormalities [6]. Increased oxidative stress may simultaneously damage erythrocyte membranes and shorten red blood cell survival [92,93]. Morphological abnormalities, including anisocytosis and poikilocytosis, have also been observed, indicating disturbances in erythrocyte development and function [6].

Aluminium-induced oxidative stress may further promote haematological dysfunction through oxidative damage to plasma proteins and blood cell membranes [92,93]. These alterations may impair oxygen transport, promote inflammatory processes, and negatively affect overall physiological function. As with skeletal toxicity, however, the strongest clinical evidence concerns individuals with high exposure or impaired renal elimination, while the haematological consequences of typical long-term dietary exposure remain less clearly defined [93].

### 7.4. Immune Effects

The immune system may also be affected by aluminium exposure [91,96]. Depending on the dose, duration and route of exposure, as well as individual susceptibility, aluminium can influence both the activation and suppression of immune responses [96]. Proposed mechanisms include chronic inflammatory signalling, altered immune cell activity, and disturbances in cytokine production [8,92,97].

Experimental studies suggest that prolonged aluminium exposure may alter lymphocyte subpopulations and disturb the balance between immune regulatory pathways. Reductions in helper and cytotoxic T lymphocytes, together with impaired phagocytic activity, have also been observed [96]. These findings indicate that aluminium may modulate both cellular and innate immune responses, although their relevance to chronic low-level dietary exposure in humans remains uncertain.

Potential associations between aluminium exposure and autoimmune phenomena have also been discussed, particularly in relation to aluminium-containing adjuvants [96]. However, these exposure scenarios differ substantially from dietary exposure in terms of route, dose, and biological context. Therefore, findings concerning aluminium adjuvants should not be directly extrapolated to aluminium consumed with food or drinking water. Proposed mechanisms involve persistent immune stimulation and low-grade inflammatory responses, but the causal relationships remain incompletely understood [8,96].

Overall, the evidence for immunological effects of aluminium is derived predominantly from experimental studies and from exposure contexts that differ substantially from chronic dietary exposure. Although aluminium can modulate inflammatory and immune signalling under experimental conditions, the relevance of these findings to aluminium ingested with food and drinking water remains uncertain [8,91,96]. In particular, evidence derived from aluminium-containing adjuvants should not be considered directly representative of dietary exposure because of substantial differences in aluminium formulation, route of administration, and systemic bioavailability [8,96]. Consequently, current evidence supports the biological plausibility of aluminium-induced immune modulation, but remains insufficient to establish clinically relevant immune effects resulting from typical chronic dietary aluminium exposure in the general population [8,91,96].

### 7.5. Metabolic and Gastrointestinal Effects

The gastrointestinal tract represents the first major biological interface with aluminium derived from food and drinking water [8,91]. Increasing experimental evidence indicates that chronic aluminium exposure may alter the composition and function of the intestinal microbiota [91]. Reported changes include reduced microbial diversity and decreases in bacterial populations considered beneficial to host health. Such alterations may impair intestinal barrier integrity and potentially increase susceptibility to chronic inflammatory processes. The interactions between dietary aluminium, intestinal microbiota, barrier function, and the gut–brain axis are discussed in greater detail in Section 8 [91,92].

Aluminium exposure has also been associated with disturbances in carbohydrate metabolism and glucose homeostasis [93]. Experimental studies have reported pancreatic β-cell damage, insulin resistance, and reduced expression of glucose transporters in peripheral tissues. These alterations may impair cellular glucose utilisation, with potential consequences for broader metabolic function. However, evidence directly linking typical levels of dietary aluminium exposure with clinically relevant metabolic disease in humans remains limited [6,93].

The liver represents another potential target of aluminium toxicity. Experimental accumulation of aluminium in hepatocytes has been associated with oxidative stress, mitochondrial dysfunction, and reduced activity of enzymes involved in energy metabolism [6]. These changes have been linked experimentally to hepatic steatosis, disturbances of the tricarboxylic acid cycle, and reduced ATP production. Persistent metabolic disruption may consequently impair hepatic and systemic metabolic function [6,91,92].

Overall, current evidence indicates that aluminium has the potential to affect multiple physiological systems through interconnected mechanisms. Nevertheless, a substantial proportion of these findings derives from experimental models, occupational exposure, dialysis-associated toxicity, or other high-exposure scenarios. Further studies are therefore required to determine whether long-term dietary aluminium exposure at levels encountered by the general population is sufficient to produce clinically significant systemic effects.

Given the substantial heterogeneity in study designs, exposure conditions, and investigated outcomes, the strength and relevance of the available evidence vary considerably across the biological mechanisms and health effects discussed above. To facilitate a critical interpretation of these findings, Table 2 summarises the predominant evidence base, consistency of findings, major methodological limitations, relevance to chronic dietary exposure, and the overall interpretation of the evidence for the principal biological mechanisms and health outcomes associated with aluminium exposure.

## 8. Dietary Aluminium, Gut Microbiota, and the Gut–Brain Axis

The gastrointestinal tract represents the primary site of contact with aluminium derived from food and drinking water. Experimental evidence suggests that aluminium exposure may alter the composition and metabolic activity of the gut microbiota, promote dysbiosis, and impair intestinal barrier integrity, potentially affecting immune and nervous system function [98,99,100,101,102,103,104].

### 8.1. Effects of Dietary Aluminium on Gut Microbiota

Experimental aluminium exposure has been associated with alterations in intestinal microbial composition, including reductions in potentially beneficial taxa and increases in microorganisms associated with dysbiosis and inflammatory processes [100,101,105,106,107]. Reported changes include reductions in Actinobacteria, Verrucomicrobia, and *Akkermansia muciniphila*, together with increased abundance of Firmicutes and Proteobacteria [98,100,101,105,107]. These alterations may affect intestinal homeostasis and barrier function.

Aluminium exposure may also modify microbial metabolism, including reduced production of short-chain fatty acids such as acetate, propionate, and butyrate, which contribute to intestinal homeostasis, immune regulation, and blood–brain barrier integrity [101,106,108,110,111]. In addition, aluminium oxide nanoparticles have been reported to facilitate horizontal transfer of antibiotic-resistance genes through increased microbial membrane permeability and oxidative stress. However, this finding relates specifically to nanoparticulate exposure and should not be extrapolated to all dietary forms of aluminium [101].

### 8.2. Intestinal Barrier Dysfunction and Systemic Inflammation

Aluminium-associated dysbiosis may contribute to impaired intestinal barrier integrity. Experimental studies have reported alterations in tight-junction proteins, including zonula occludens-1, claudin-1, and occludin, which may increase intestinal permeability and facilitate the translocation of bacterial lipopolysaccharides, microbial metabolites, and other potentially harmful molecules into the systemic circulation [98,108,109,110,111,112,113,114,115]. These changes may enhance oxidative stress and promote the production of pro-inflammatory cytokines, including TNF-α, IL-1β, and IL-6, thereby contributing to low-grade systemic inflammation [104,109,110,112]. However, evidence for this pathway is predominantly experimental, and its relevance to chronic dietary aluminium exposure in humans remains insufficiently established.

### 8.3. Gut–Brain Axis and Potential Neurological Consequences

Aluminium-induced alterations in the gut microbiota and intestinal barrier may potentially influence central nervous system function through the gut–brain axis [113,114]. Increased intestinal permeability may facilitate systemic exposure to microbial products and inflammatory mediators, whereas reduced production of short-chain fatty acids may adversely affect blood–brain barrier integrity and microglial regulation [101,105,106,108,109,112,113,116,117,118]. Together, these processes provide a plausible indirect pathway linking gastrointestinal aluminium exposure with neuroinflammatory responses.

Experimental studies have also suggested that aluminium-associated dysbiosis may contribute to processes relevant to neurodegeneration, including oxidative stress, neuroinflammation, β-amyloid deposition, and tau hyperphosphorylation [88,105,106,111,114,117,119,120]. However, these findings remain predominantly mechanistic and preclinical. Current evidence does not establish the gut microbiota as a causal mediator between typical dietary aluminium exposure and Alzheimer’s disease or other neurodegenerative disorders.

### 8.4. Potential Microbiota-Targeted Mitigation Strategies

Experimental evidence suggests that modulation of the gut microbiota may represent a potential strategy for mitigating some biological effects associated with aluminium exposure [98,114]. *Lactobacillus plantarum* and *Akkermansia muciniphila* have been investigated for their potential to bind aluminium within the intestinal lumen, reduce its gastrointestinal absorption, and support microbial and intestinal barrier homeostasis [102,114,115,121]. In animal models, microbiota-targeted interventions have also been associated with reductions in oxidative and inflammatory responses and improvements in cognitive performance [102,114,115,119]. However, these findings remain predominantly preclinical, and there is currently insufficient human evidence to recommend probiotic or other microbiota-targeted interventions for preventing or reducing adverse effects associated with chronic dietary aluminium exposure.

## 9. Global Dietary Exposure and Health Risk Assessment

Global assessments of aluminium exposure indicate substantial variation in dietary intake across geographical regions and population groups [34,122]. The magnitude of exposure is determined primarily by local dietary patterns, the types of foods consumed, the use of aluminium-containing food additives, food-processing practices, and, to some extent, environmental contamination [122,123]. Available data indicate that dietary aluminium exposure in certain populations may approach or exceed established health-based guidance values [34,122].

Health risk assessment is primarily based on comparing estimated dietary aluminium intake with reference values established by international regulatory and scientific organisations [122,124]. Particular attention should therefore be directed toward identifying population groups with the highest exposure and determining the foods that contribute most substantially to total aluminium intake [124,125]. Although food represents the dominant exposure source for the general population, drinking water should also be considered when estimating overall oral exposure, particularly in regions where aluminium concentrations are elevated [34,123].

### 9.1. Regional Patterns and Selected Examples of Dietary Aluminium Intake

Studies conducted across different geographical regions demonstrate considerable variation in dietary aluminium exposure [122]. However, the quantitative examples presented in this section are based primarily on available total-diet studies from selected East Asian populations, including Hong Kong, Shenzhen, and Taiwan [122,124,125]. These populations are used as illustrative regional examples and should not be interpreted as representative of global dietary aluminium exposure. The limited geographical coverage of directly comparable quantitative data highlights the need for harmonised exposure assessments from a broader range of regions. In Hong Kong, aluminium has been identified as an important food contaminant, with cereal products and beverages representing major contributors to overall dietary exposure [122]. In Shenzhen, particularly high aluminium concentrations have been reported in certain food categories, including jellyfish products and fried flour-based foods [125]. Similarly, in Taiwan, aluminium exposure has been associated with the consumption of certain noodle and cereal-based products in which aluminium-containing compounds may be introduced during food processing [124].

In European countries, aluminium exposure is also subject to food safety assessment and regulatory monitoring, although dietary risk assessments frequently consider it alongside other potentially harmful elements and contaminants, including lead, cadmium, and inorganic arsenic [122,123]. The available evidence nevertheless indicates that aluminium remains relevant to food safety because of its widespread occurrence in foods and the possibility of relatively high exposure among specific consumer groups [122].

Regional differences in aluminium exposure primarily reflect variations in dietary habits, consumption patterns, food-processing technologies, and the regulatory use of aluminium-containing additives [122,124]. Consequently, dietary exposure may differ substantially between populations, even among regions with comparable levels of socioeconomic development. These findings highlight the importance of population-specific exposure assessments rather than relying exclusively on generalised global intake estimates [34,122,124].

### 9.2. Major Dietary Contributors and Levels of Aluminium Intake

As a general reference point, EFSA estimated mean dietary aluminium exposure among European adults at approximately 1.6–13 mg/day, equivalent to approximately 0.2–1.5 mg/kg body weight/week [26]. Available exposure assessments demonstrate substantial differences in estimated aluminium intake between populations [122]. In Hong Kong, mean weekly dietary exposure has been estimated at approximately 0.600 mg/kg body weight [122]. Higher mean weekly exposures have been reported in Shenzhen (1.263 mg/kg body weight) [125] and Taiwan (1.071 mg/kg body weight) [124]. Both estimates exceed the EFSA TWI of 1 mg/kg body weight per week, corresponding to approximately 126.3% and 107.1% of the TWI, respectively, whereas the Hong Kong estimate remains below this value. Selected regional exposure estimates and their comparison with the EFSA TWI and JECFA PTWI are summarized in Table 3. These estimates are intended to provide illustrative examples of population-level exposure rather than a comprehensive global comparison.

The principal dietary contributors vary according to regional consumption patterns. Frequently identified sources include cereal products, fried flour-based foods, leafy vegetables, legumes, beverages, and selected seafood products [122,125]. Particularly high aluminium concentrations have been reported in certain fried cereal products and processed jellyfish. Regular consumption of such foods may therefore contribute disproportionately to total dietary exposure [122,125].

The contribution of individual food categories to overall exposure depends not only on their aluminium concentration but also on the frequency and quantity of consumption. Foods containing moderate aluminium concentrations may therefore become major contributors when consumed regularly, whereas products with exceptionally high concentrations may have a relatively limited impact if consumed only occasionally.

At the individual level, dietary composition can therefore modify total aluminium intake by several-fold. As a broad quantitative reference, EFSA estimated mean dietary aluminium exposure among European adults at approximately 1.6–13 mg/day, representing an approximately eight-fold range [26]. This variability cannot be attributed exclusively to diet composition, because analytical methods, regional food contamination, food processing, and other factors also contribute; nevertheless, differences in the habitual consumption of aluminium-rich foods are an important determinant of individual exposure [16,26,122]. Regular consumption of tea, cocoa products, certain herbs and spices, cereal- and flour-based products, or foods containing aluminium-based additives may substantially increase total intake compared with diets dominated by foods naturally containing aluminium in the low mg/kg range [16,34,122,125]. Consequently, two individuals consuming similar quantities of food may experience markedly different aluminium exposure depending on the types and processing characteristics of the foods selected.

In clinical practice, conventional dietary questionnaires or food-frequency questionnaires may provide a qualitative or semi-quantitative indication of dietary aluminium exposure by identifying frequent consumption of major aluminium-contributing food categories. However, their ability to quantify individual aluminium intake accurately is limited. Aluminium concentrations may vary substantially within the same food category according to geographical origin, environmental contamination, processing methods, use of aluminium-containing additives, preparation practices, and contact with aluminium-containing materials [16,26,34,122]. Consequently, assigning a single aluminium concentration to a food item reported in a conventional dietary questionnaire may introduce considerable exposure misclassification. More reliable quantitative assessment requires combining detailed individual food-consumption data with representative analytical measurements of aluminium concentrations in the foods actually consumed, as performed in dietary exposure and total-diet studies [16,26,122,124,125]. Therefore, conventional clinical dietary questionnaires may be useful for identifying potentially high-exposure dietary patterns but should not be regarded as validated stand-alone tools for precise estimation of individual aluminium intake.

Drinking water generally contributes less to total aluminium intake than food. Nevertheless, its universal and continuous consumption makes it a relevant component of overall oral exposure. Comprehensive dietary risk assessments should therefore consider aluminium intake from both food and drinking water, while clearly distinguishing these sources from non-dietary exposure routes [122,124,125].

### 9.3. Comparison with Health-Based Guidance Values

Assessment of the potential health risks associated with dietary aluminium exposure relies on comparison with health-based guidance values established by international scientific and regulatory bodies [34,122,123]. In 2011, the Joint FAO/WHO Expert Committee on Food Additives (JECFA) established a provisional tolerable weekly intake (PTWI) of 2 mg/kg body weight per week, based on a no-observed-adverse-effect level (NOAEL) of 30 mg/kg body weight per day and the application of an uncertainty factor of 100 [123]. In 2008, the European Food Safety Authority (EFSA) established a tolerable weekly intake (TWI) of 1 mg/kg body weight per week based on the combined evidence from animal studies showing adverse effects on the developing nervous system, reproductive system, and other toxicological endpoints [26].

This value has been widely applied in European dietary exposure assessments and provides an important benchmark for evaluating long-term aluminium intake [122]. The reported regional weekly dietary aluminium exposure estimates and their relation to the EFSA TWI and JECFA PTWI are summarized in Table 3.

Comparison of the reported regional exposure estimates with these health-based guidance values indicates that mean dietary aluminium exposure may exceed the EFSA TWI in some populations (Table 3). The estimated mean weekly exposures reported for Shenzhen (1.263 mg/kg bw/week) and Taiwan (1.071 mg/kg bw/week) correspond to approximately 126.3% and 107.1% of the EFSA TWI, respectively, whereas the estimate for Hong Kong (0.600 mg/kg bw/week) corresponds to 60.0% of the TWI. In contrast, all three mean exposure estimates remain below the JECFA PTWI of 2 mg/kg bw/week. These comparisons demonstrate that risk characterisation may differ according to the health-based guidance value applied and highlight the importance of considering regional differences in dietary aluminium exposure [122,124,125].

Risk characterisation should nevertheless extend beyond direct comparison of mean population intake with a single guidance value. High-percentile consumers may experience substantially greater exposure than the population average, particularly when their diets contain frequently consumed foods with elevated aluminium concentrations. Furthermore, because the gastrointestinal bioavailability of aluminium varies according to chemical form and food matrix, total dietary intake does not necessarily correspond directly to the systemically absorbed dose.

### 9.4. Quantitative Risk Characterisation and Uncertainty

To provide a quantitative characterisation of dietary aluminium exposure, both mean and high-percentile (P95) weekly exposure estimates, where available, were considered for comparison with the health-based guidance values established by EFSA and JECFA. The RCR was calculated as the ratio of estimated weekly dietary exposure (EWE) to the corresponding tolerable weekly intake (TWI or PTWI): RCR = EWE/HBGV. An RCR below 1 indicates that the estimated exposure remains below the respective health-based guidance value, whereas an RCR above 1 indicates that the guidance value is exceeded. This ratio should not be interpreted as a direct probability or magnitude of adverse health effects, but rather as a screening-level measure of the extent to which estimated population exposure approaches or exceeds the respective guidance value [34,122,123].

Using the EFSA TWI of 1 mg/kg bw/week, the calculated RCRs were 0.600 for Hong Kong, 1.263 for Shenzhen, and 1.071 for Taiwan [122,124,125]. Thus, mean exposure estimates for Shenzhen and Taiwan exceeded the EFSA guidance value, whereas the Hong Kong estimate remained below it. When the JECFA PTWI of 2 mg/kg bw/week was used as the reference value, the corresponding RCRs were 0.300, 0.632, and 0.536, respectively, and none of the mean exposure estimates exceeded the JECFA PTWI [34,122,123,124,125]. These results demonstrate that quantitative risk characterisation is sensitive to the health-based guidance value selected and that the same exposure estimate may lead to different screening-level interpretations depending on the regulatory benchmark applied.

Several sources of uncertainty should be considered when interpreting the calculated RCRs [122,124,125]. First, comparisons between regional estimates are affected by differences in dietary assessment methods, food-consumption patterns, analytical procedures, food categorisation, and the aluminium concentrations assigned to individual food items, which may contribute to exposure misclassification and limit direct comparability between populations [122,124,125]. Second, analytical comparability represents an additional source of uncertainty because differences in sample preparation, digestion procedures, analytical techniques, detection limits, quality-control protocols, and the treatment of values below the limit of detection may influence reported aluminium concentrations. Consequently, quantitative comparisons between studies using different analytical methodologies should be interpreted with caution [122,124,125]. Third, estimates of total dietary aluminium intake do not account for variation in gastrointestinal bioavailability associated with aluminium speciation, food-matrix composition, nutritional status, and interactions with dietary constituents [18,19,20,26]. Consequently, similar external dietary exposures may not result in equivalent internal aluminium doses. Finally, the use of different health-based guidance values introduces additional interpretative uncertainty, as demonstrated by the different RCR classifications obtained using the EFSA TWI and JECFA PTWI [34,122,123]. Accordingly, risk assessment in the present review is restricted to exposure characterisation and comparison with established health-based guidance values and should not be interpreted as a comprehensive quantitative health risk assessment.

### 9.5. Vulnerable Population Groups

Dietary aluminium exposure is not distributed uniformly across the population [125]. Children represent an important group in dietary exposure assessments because their lower body weight can result in substantially higher aluminium intake when expressed per kilogram of body weight [122]. Their dietary patterns may also include frequent consumption of processed snacks, bakery products, and flour-based foods that can contribute to aluminium exposure [34]. Infants and young children may be particularly susceptible because aluminium intake per kilogram of body weight can be comparatively high during early life. Quantitative estimates further demonstrate the importance of exposure during this developmental period. EFSA estimated weekly aluminium exposure in infants aged 0–3, 4–6, 7–9, and 10–12 months at approximately 0.10, 0.20, 0.43, and 0.78 mg/kg bw/week, respectively. For 3-month-old infants, estimated exposure from infant formula reached up to 0.6 mg/kg bw/week for milk-based formula and 0.75 mg/kg bw/week for soya-based formula at mean exposure, increasing to approximately 0.9 and 1.1 mg/kg bw/week, respectively, at high-percentile exposure [26]. These findings support specific consideration of infants and young children in dietary aluminium risk assessment rather than extrapolation from adult exposure estimates.

Pregnant women and women of reproductive age also warrant consideration in population-based exposure assessments [34,124]. Studies conducted in Asian populations have indicated that dietary exposure to food contaminants may be elevated in subsets of women of reproductive age [124]. However, aluminium-specific risks during pregnancy should be interpreted cautiously unless exposure data and health outcomes have been directly assessed, as evidence concerning the consequences of typical dietary exposure for fetal development remains limited [122].

Older adults may represent another potentially vulnerable population [124]. In Hong Kong, relatively high aluminium exposure has been reported among men aged 70–84 years, which was attributed primarily to specific dietary consumption patterns. In addition to dietary habits, age-related reductions in renal function may theoretically influence aluminium elimination and long-term retention, making total exposure particularly relevant in older individuals [15].

Socioeconomic factors may also contribute to differences in exposure by influencing food choices, dietary diversity, environmental conditions, and access to less processed foods. However, evidence directly demonstrating socioeconomic inequalities specifically in aluminium exposure remains limited and should not be generalised beyond populations in which such associations have been investigated [122,124].

## 10. Discussion

The available evidence reveals a marked discrepancy between the well-established toxicological potential of aluminium under conditions of elevated exposure and the considerably greater uncertainty surrounding chronic exposure at levels typically encountered through the diet. High-dose experimental studies and clinical observations from historically exposed populations provide consistent evidence that aluminium can accumulate in tissues and exert adverse biological effects. In contrast, extrapolation of these findings to the general population is constrained by the very low gastrointestinal absorption of dietary aluminium, substantial variation in aluminium speciation and bioavailability, and the limited availability of prospective human studies directly linking quantified dietary exposure with long-term health outcomes. Consequently, hazard identification for aluminium is considerably better established than characterisation of the health risk associated with usual dietary exposure.

The heterogeneity of the available evidence also limits formal quantitative synthesis. Differences in aluminium species, exposure route, dose and duration, study population, biological matrices, and outcome definitions prevent direct pooling of most mechanistic and health-effect studies into a single meaningful effect estimate. Accordingly, the quantitative component of the present review focuses on directly comparable dietary exposure estimates and their relationship to established health-based guidance values, while evidence concerning biological mechanisms and health outcomes is synthesised through structured appraisal of consistency, methodological limitations, and relevance to chronic dietary exposure. This distinction is important because statistical pooling of fundamentally heterogeneous exposure scenarios could produce a quantitatively precise but biologically misleading summary estimate.

The mechanistic literature provides biological plausibility for aluminium toxicity, but the strength of evidence is not uniform across the proposed pathways. Oxidative stress, mitochondrial dysfunction, disturbances in mineral homeostasis, and inflammatory responses are supported by multiple experimental studies, whereas more specific molecular mechanisms remain less consistently demonstrated. Importantly, much of this evidence originates from in vitro systems, animal models, or exposure conditions exceeding those expected from a typical human diet. This limits direct translation of mechanistic findings into estimates of disease risk in the general population. The discrepancy is particularly relevant to neurotoxicity: although experimental evidence supports several pathways through which aluminium could adversely affect neuronal function, their clinical significance under chronic dietary exposure remains uncertain, including in the context of the neurodegenerative outcomes discussed in Section 7.1. The gut microbiota–gut–brain axis represents an emerging component of the evidence base. Although experimental studies provide mechanistic plausibility, the evidence remains dominated by animal and in vitro models, and human studies directly linking quantified dietary aluminium exposure with microbiota alterations and subsequent systemic or neurological outcomes are lacking. Consequently, this pathway cannot yet be considered an established mediator of the health effects of dietary aluminium.

From a risk-assessment perspective, dietary aluminium exposure cannot be characterised uniformly across populations. Although average intake frequently remains below established health-based guidance values, selected populations and high consumers may approach or exceed the EFSA TWI. Such exceedance should be interpreted as a screening-level indication of reduced safety margin rather than evidence of adverse health effects. Methodological heterogeneity between exposure studies further limits direct regional comparisons and highlights the need for harmonised dietary exposure assessments.

Several evidence gaps currently prevent a more definitive assessment of the health significance of chronic dietary aluminium exposure. First, prospective human studies combining repeated dietary exposure assessment with biomarkers of internal aluminium exposure and long-term health outcomes are scarce. Second, total aluminium intake alone may inadequately characterise biologically relevant exposure because gastrointestinal absorption varies with aluminium speciation, food matrix composition, nutritional status, and interactions with dietary constituents. Third, experimental studies frequently employ aluminium compounds, doses, and exposure routes that differ substantially from those encountered through habitual human diets, limiting their direct translational relevance. Fourth, evidence concerning microbiota-mediated effects and the gut–brain axis remains predominantly experimental, with a lack of longitudinal human studies integrating dietary aluminium exposure, microbiome composition, intestinal barrier function, and neurological outcomes. Addressing these evidence gaps will be essential for determining the health relevance of chronic dietary aluminium exposure under real-world nutritional conditions.

## 11. Conclusions

Dietary aluminium exposure is highly variable and is determined by both the aluminium content of consumed foods and factors affecting its gastrointestinal bioavailability. Although only a small fraction of ingested aluminium is absorbed, prolonged exposure and impaired elimination may increase systemic aluminium burden, particularly in susceptible populations. Current evidence supports the toxicological potential of aluminium under elevated-exposure conditions, but does not demonstrate that typical dietary exposure causes chronic disease in the general population. From a food-safety perspective, the principal priority is therefore the identification and monitoring of population groups with persistently elevated intake, together with improved characterisation of the bioavailable aluminium dose rather than solely the total aluminium dose.

## Figures and Tables

**Table 1:** Aluminium-containing food additives, their E-numbers, principal technological roles, and current regulatory status in the European Union.

E-Number	Food Additive	Principal Technological Role/Authorised Use	Current EU Status
E173	Aluminium	Food colour; currently authorised only for restricted decorative/external coating uses in confectionery	Authorised with strict restrictions
E520	Aluminium sulphate	Firming/stabilising applications; currently permitted in highly restricted uses, including candied cherries and liquid egg white for egg foams	Authorised with strict restrictions
E521	Aluminium sodium sulphate	Aluminium sulphate-group additive; historically used mainly for firming/stabilising purposes	Authorised only within restricted E520–E523 uses
E522	Aluminium potassium sulphate	Aluminium sulphate-group additive; stabilising/firming applications	Authorised only within restricted E520–E523 uses
E523	Aluminium ammonium sulphate	Aluminium sulphate-group additive; firming/stabilising applications	Authorised only within restricted E520–E523 uses
E541	Sodium aluminium phosphate, acidic	Leavening/raising agent in selected bakery products	Authorised with strict restrictions
E554	Sodium aluminium silicate	Anti-caking/carrier-related use; currently allowed only in specific technological applications	Authorised with strict restrictions
E555	Potassium aluminium silicate	Carrier, including use in preparations of iron oxides and hydroxides (E172)	Authorised with restricted use
E556	Calcium aluminium silicate	Former anti-caking/carrier-type aluminium silicate	No longer authorised for food use in the EU
E558	Bentonite	Former processing/anti-caking-type silicate additive	No longer authorised for food use in the EU
E559	Aluminium silicate (kaolin)	Former anti-caking/carrier-type additive	No longer authorised for food use in the EU

**Table 2:** Critical appraisal of the evidence concerning the biological mechanisms and potential health effects of aluminium exposure.

Evidence Domain	Predominant Evidence Base	Consistency of Evidence	Major Limitations	Relevance to Chronic Dietary Exposure	Overall Interpretation	Ref.
Oxidative stress and mitochondrial dysfunction	Predominantly in vitro and animal studies	Relatively consistent experimental findings	Experimental doses and exposure conditions frequently differ from habitual dietary exposure; limited direct human evidence	Limited to moderate	Well-supported mechanistic pathway of aluminium toxicity, but its magnitude under typical dietary exposure in humans remains uncertain	[6,23,73,74,75,76,77,78,79,80,81]
Mineral homeostasis	Experimental studies, supported by toxicokinetic evidence	Generally consistent	Dose-, tissue-, and exposure-dependent effects; limited confirmation under usual human dietary exposure	Limited to moderate	Biologically plausible and experimentally supported mechanism, particularly at elevated exposure	[6,21,63,73,74,75,76,77,78,79,80]
Inflammatory and immune effects	Predominantly experimental studies; selected human evidence from non-dietary exposure contexts	Variable	Heterogeneous aluminium forms and exposure routes; evidence from adjuvants and high-exposure settings cannot be directly extrapolated to dietary exposure	Limited	Immune and inflammatory modulation is biologically plausible, but clinically relevant effects from typical dietary exposure remain insufficiently established	[8,23,81,88,91,92,96,97]
Neurotoxicity	Experimental studies and human evidence from high-exposure/clinical settings	Relatively consistent for high exposure; less consistent for low-level exposure	Major differences in dose, route, aluminium species, and exposure duration; limited prospective dietary studies	Moderate for hazard identification; limited for usual dietary risk	Neurotoxic potential is established under elevated systemic exposure, whereas effects of typical chronic dietary exposure remain uncertain	[6,8,23,75,81,85,91,92,93]
Alzheimer’s disease and neurodegeneration	Experimental and observational evidence	Inconsistent	Residual confounding, heterogeneous exposure assessment, uncertain temporality, and limited ability to establish causality	Limited	Mechanistic plausibility exists, but a causal relationship between typical dietary aluminium exposure and Alzheimer’s disease has not been established	[6,8,91,93]
Metabolic and gastrointestinal effects	Predominantly experimental evidence	Emerging/variable	Limited human evidence and frequent use of exposure levels not representative of habitual diets	Limited	Potential metabolic and gastrointestinal effects are biologically plausible but remain insufficiently characterised in humans	[6,91,92,93]
Gut microbiota and gut–brain axis	Predominantly animal and in vitro studies	Emerging	Scarce direct human evidence; differences in aluminium species, dose, food matrix, and experimental models	Limited	Emerging mechanistic pathway that cannot yet be considered an established mediator of chronic dietary aluminium effects	[98,99,100,101,102,103,104,105,106,107,108,109,110,111,112,113,114,115]

**Table 3:** Comparison of reported regional mean and high-percentile weekly dietary aluminium exposure with the EFSA TWI and JECFA PTWI.

Region/Population	Mean Weekly Al Intake (mg/kg bw/Week)	EFSA TWI (mg/kg bw/Week)	RCR (EFSA), Mean	JECFA PTWI (mg/kg bw/Week)	RCR (JECFA), Mean	P95 Weekly Al Intake (mg/kg bw/Week)	RCR (EFSA), P95	RCR (JECFA), P95	Interpretation	Reference
Hong Kong	0.600	1.0	0.600	2.0	0.300	1.500	1.500	0.750	Mean below both HBGVs; P95 exceeds EFSA TWI but remains below JECFA PTWI	[122]
Shenzhen, China	1.263	1.0	1.263	2.0	0.632	4.720	4.720	2.360	Mean exceeds EFSA TWI but remains below JECFA PTWI; P95 exceeds both HBGVs	[125]
Taiwan	1.071	1.0	1.071	2.0	0.536	NR	NR	NR	Mean exceeds EFSA TWI but remains below JECFA PTWI; population-wide P95 not reported	[124]

RCR, risk characterization ratio; TWI, tolerable weekly intake; PTWI, provisional tolerable weekly intake; HBGV, health-based guidance value; P95, 95th percentile; NR, not reported. RCR was calculated as estimated weekly dietary aluminium exposure divided by the corresponding HBGV. An RCR > 1 indicates that the respective HBGV is exceeded.

## Data Availability

No new data were created or analyzed in this study. Data sharing is not applicable to this article.
